# Clinical results of treatment using a modified K-wire tension band versus a cannulated screw tension band in transverse patella fractures: A strobe-compliant retrospective observational study

**DOI:** 10.1097/MD.0000000000004992

**Published:** 2016-10-07

**Authors:** Honglue Tan, Pengyi Dai, Yanhao Yuan

**Affiliations:** Luoyang Orthopedics and Traumatology Institution, Luoyang Orthopedic-Traumatological Hospital, Luoyang Henan, China.

**Keywords:** cannulated screw tension band, modified K-wire tension band, patellar fracture, surgical treatment

## Abstract

It was a retrospective case–control study. The aim of this study was to explore the clinical efficacy and complication of treatment using a modified Kirschner wire tension band (MKTB) or a cannulated screw tension band (CSTB) in transverse patellar fractures.

In total, 55 patients with transverse patellar fractures were retrospectively reviewed and divided into 2 groups according to the surgical technique: 29 patients were in the MKTB group and 26 patients in the CSTB group. Bǒstman's clinical grading scale, including range of movement (ROM), pain, ability to work, atrophy of quadriceps femoris, assistance in walking, effusion, giving way, and stair-climbing, was used to evaluate the clinical results. Complications including painful hardware, implant loosening or breakage, and bone nonunion were also assessed.

Both groups were evaluated at the final follow-up before removing implant in the MKTB group. The Bǒstman's score of ROM, pain, atrophy of quadriceps femoris, and effusion were all higher in the CSTB group than in the MKTB group (*P* < 0.05). Twelve patients in the MKTB group underwent implant removal, and the score of ROM, pain, and effusion were higher than before removing implant (*P* < 0.05), but there was no difference compared to the CSTB group (*P* > 0.05). Seventeen patients achieved excellent results, 9 had good results, and 3 reported fair results in the MKTB group; the CSTB group had excellent results in 22 patients and good results in 4 patients, showing a significant difference in the excellent rate between the 2 groups (*P* = 0.021). Total Bǒstman scores in the MKTB and CSTB groups (26.96 ± 4.47 and 29.42 ± 1.47, respectively) were significantly different (*P* = 0.01). Total scores in the MKTB group after removing implant were higher than those before removing implant (*P* = 0.001), and similar to those in the CSTB group (*P* *=* 0.224). Eleven patients in the MKTB group reported painful hardware, including 4 cases of implant loosening.

CSTB achieves better clinical results than MKTB, meanwhile avoiding the problems of painful hardware and implant loosening. Functional limitation caused by hardware pain was commonly seen in the MKTB group, and removing implant after fracture healing improved knee function.

## 1 Introduction

Open reduction and internal fixation is presently the preferred method for treatment of displaced patellar fractures, with the aim of restoring the normal patellar configuration and integration of the knee extensor mechanism by reliable internal fixation, which allows for early rehabilitation of the knee joint.^[1,2]^ The modified Kirschner wire tension band (MKTB) involving longitudinal Kirschner wires (K-wires) and steel wire in a figure-of-eight pattern looped over the anterior patella is the most widely accepted method for transverse patellar fractures, which converts the anterior tension forces produced by the extensor mechanism and knee flexion into compression forces at the articular surface.^[2]^ However, postoperative complications of MKTB including symptomatic hardware, implant displacement, loss of reduction, and displacement of fracture fragments have also been documented.^[2,3]^ These complications often require a second surgical procedure for removal of the implants. Therefore, techniques employing alternatives to MKTB have been investigated.

Screws have recently been used for internal fixation of transverse patella fractures, which show better biomechanical stabilization than K-wires by resisting tensile loading throughout knee flexion.^[2]^ Biomechanical testing in cadaveric transverse patella fractures has shown that cancellous screws combined with a tension band had better biomechanical performance than a traditional modified anterior tension band (MATB).^[4]^ In the clinical setting, however, it is usually difficult to roll the tension band around the tips of the screws for treatment of patella fractures. Thus, a new method with a figure-of-eight tension band wire passing through 2 parallel cannulated screws was designed to overcome this problem. Additional cadaveric mechanical testing showed that the cannulated lag screws with the tension band passing through the screws had stronger fixation strength than screws alone or a modified tension band for transverse patella fractures.^[5]^ Tian et al^[6]^ found that a titanium cable-cannulated screw tension band can achieve good fracture reduction and healing with a low rate of complications.

In order to avoid the above shortcomings of the MKTB technique, we used cannulated lag screws with a wire tension band (CSTB) for the treatment of transverse patellar fractures. The purpose of this study was to compare CSTB with MKTB to see whether the former technique could achieve stable internal fixation and better knee function, and in addition reduce the rate of complications including painful hardware, implant loosening, and loss of reduction.

## 2 Patients and methods

### 2.1 Patients

The study was approved by the Ethical Review Boards of Luoyang Orthopedics and Traumatology Institution, Luoyang Orthopedic-Traumatological Hospital. All patients provided written informed consent. This study retrospectively reviewed patients with transverse patellar fractures who underwent surgical treatment between June 2012 and April 2015. The inclusion criteria were as follows: (1) transverse fractures with or without a single additional fragment caused by a longitudinal fracture line; (2) age 18 to 55 years without previous knee surgery or other fracture; (3) fresh, unilateral fractures with an obvious interfragmentary gap; (4) C1 and C2 type according to AO classification;^[7]^ (5) fixation with either an MKTB or CSTB; (6) at least a 12-month follow-up. According to these criteria, 55 patients were included in the study.

The patients were divided into MKTB and CSTB groups according to the surgical technique. The MKTB group comprised 29 patients: 22 men and 7 women. Mean age was 37.12 ± 10.35 years (range 18–54 years). The body mass index (BMI) was 22.63 ± 1.47. Seven injuries were caused by falls from height, 5 by vehicle accidents, 5 by sports injuries, and 12 by tumbling. The injured side was the right knee in 16 patients and the left in 13 patients. The fracture type was C1 in 19 patients and C2 in 10 patients. The interfragmentary gap was 15.28 ± 3.91 mm (8–22 mm). Mean operation time was 59.62 ± 7.06 minutes (range 45–74 minutes). Average follow-up time was 20.79 ± 5.36 months (range 12–36 months). Twelve patients in the MKTB group underwent implant removal because of joint pain and were followed up for a mean period of 7 ± 0.2 months (range 5–10 months).

The CSTB group comprised 26 patients: 19 men and 7 women. Mean age was 35.96 ± 10.75 years (range 18–54 years). The BMI was 22.07 ± 1.49. Six injuries were caused by falls from height, 4 by vehicle accidents, 5 by sports injuries, and 11 by tumbling. Seventeen patients had injuries in the right knee and 9 in the left knee. The fracture type was C1 in 16 patients and C2 in 10 patients. The interfragmentary gap was 15.42 ± 4.15 mm (9–22 mm). Mean operation time was 62.00 ± 6.54 minutes (range 49–75 minutes). Average follow-up time was 21.89 ± 4.72 months (range 12–28 months). Patients’ clinical data are presented in Table 1.

**Table 1:**
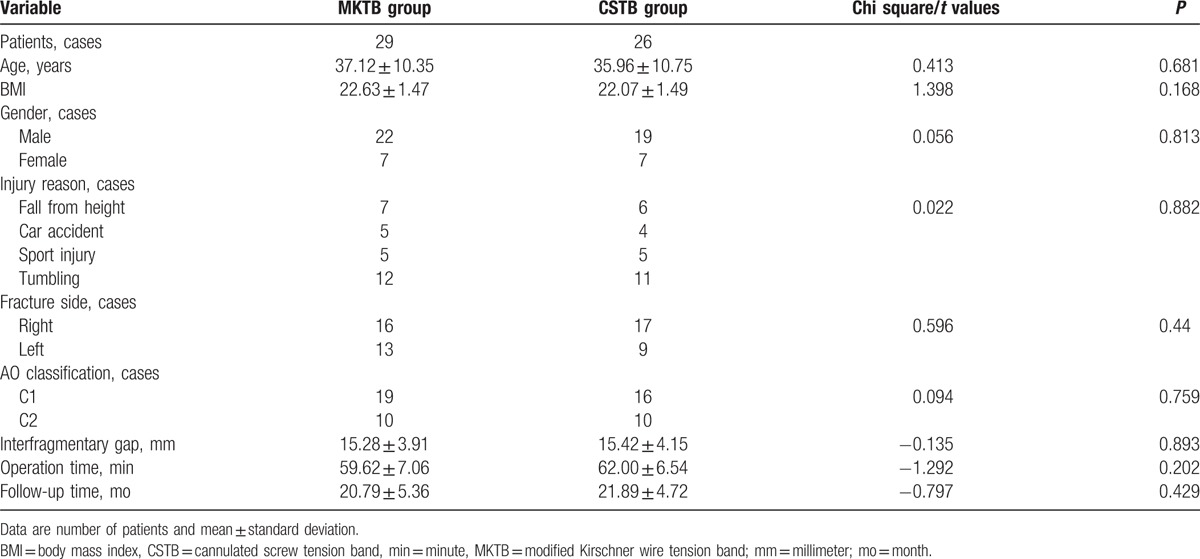
Clinical demographic data between the 2 groups.

### 2.2 Operative technique

The patient was placed in the supine position, and surgery was performed under lumbar anesthesia. An anterior longitudinal midline incision over the knee was performed in both groups, and the entire flap was sectioned until the fragments and peripatellar tissue were fully exposed. Any bone chips and hematoma were removed, and the articular cavity was rinsed with sterilized saline. The distal and posterior fragments were reduced and temporarily fixed with 2 large towel clamps. In the MKTB group, 2 parallel K-wires (2.0 mm in diameter) were drilled from the superior to the inferior pole of the patella, with a 2-cm space between the 2 wires and a distance of 10 mm from the articular surface. Steel wire (1.2 mm in diameter, 10 patients) or titanium cable (1.3 mm diameter, Zimmer Inc., Warsaw, IN; 19 patients) was threaded through the ends of the 2 K-wires to form a longitudinal figure-of-eight tension band at the anterior side of the patella, and then the steel wire was tightened using 2 forceps or the titanium cable was fixed using cable clamps. The upper end of the K-wire was bent into hooks and buried in the peripatellar tissue. If the reduction of the patellar surface was satisfactory under intraoperative fluoroscopy, the distal tails of the K-wires and any excess steel wire or titanium cable were cut off and also buried in soft tissue.

In the CSTB group, 2 parallel guide pins (1.3 mm in diameter), drilled from the superior pole to the inferior pole of the patella, were used to fix the fragments after reduction of the articular surface. The 2 pins were positioned 2 cm apart and 10 mm from the articular surface. After measuring the depth of the pins and drilling along the guide pin with a cannulated bit (2.5 mm), 2 3.2-mm titanium cannulated lag screws were threaded along the guide pin. Subsequently the pin was removed and the steel wire (1.2 mm in diameter) was passed through the cannulated screw to form a transverse figure-of-eight tension band at the anterior side of the patella, then the excess wire was cut off and buried in soft tissue.

### 2.3 Postoperative management and evaluation

None of the patients required knee immobilization postoperatively. Static quadriceps exercises, straight-leg raising, and progressive knee joint flexion were started as early as postoperative day 1. Partial-weight-bearing exercises with the help of crutches were allowed for 6 weeks postoperatively, and then the patient was permitted to fully ambulate. Anteroposterior and lateral radiographs were obtained at 2 days, and 1, 3, 6, and 12 months postoperatively. Knee function was evaluated according to the method of Bǒstman, which included range of movement (ROM, 3–6 points), pain (0–6 points), ability to work (0–4 points), atrophy of the quadriceps femoris (0–4 points), assistance in walking (0–4 points), effusion (0–2 points), giving way (0–2 points), and stair-climbing (0–2 points).^[1]^ The maximum total score is 30 points: a score of 30–28 points is excellent, 20–27 points is good, and less than 20 points is fair.^[1]^

### 2.4 Statistical analysis

Results were analyzed using the SPSS statistical software (SPSS Inc., Chicago, IL). Independent-Samples *t* test was used to determine the statistical significance of differences in measurement data between the 2 groups. Paired-Samples *t* test was used to determine the differences in measurement data before and after removing implant in the MKTB group The Chi-square test was used for analysis of enumeration data of the preoperative demographics between the 2 groups. A *P* value ≤0.05 was considered significant.

## 3 Results

### 3.1 Patient characteristics

Table 1 shows that the constituent proportions of gender, injury reason, fracture side, and AO classification were consistent between the MKTB and CSTB groups with no significant differences (*P* > 0.05). Furthermore, no significant statistical differences existed in mean age (*P* = 0.681), BMI (*P* = 0.168), interfragmentary gap (*P* = 0.893), operation time (*P* = 0.202), and follow-up time (*P* = 0.429).

### 3.2 Range of movement

**Figure 1:**
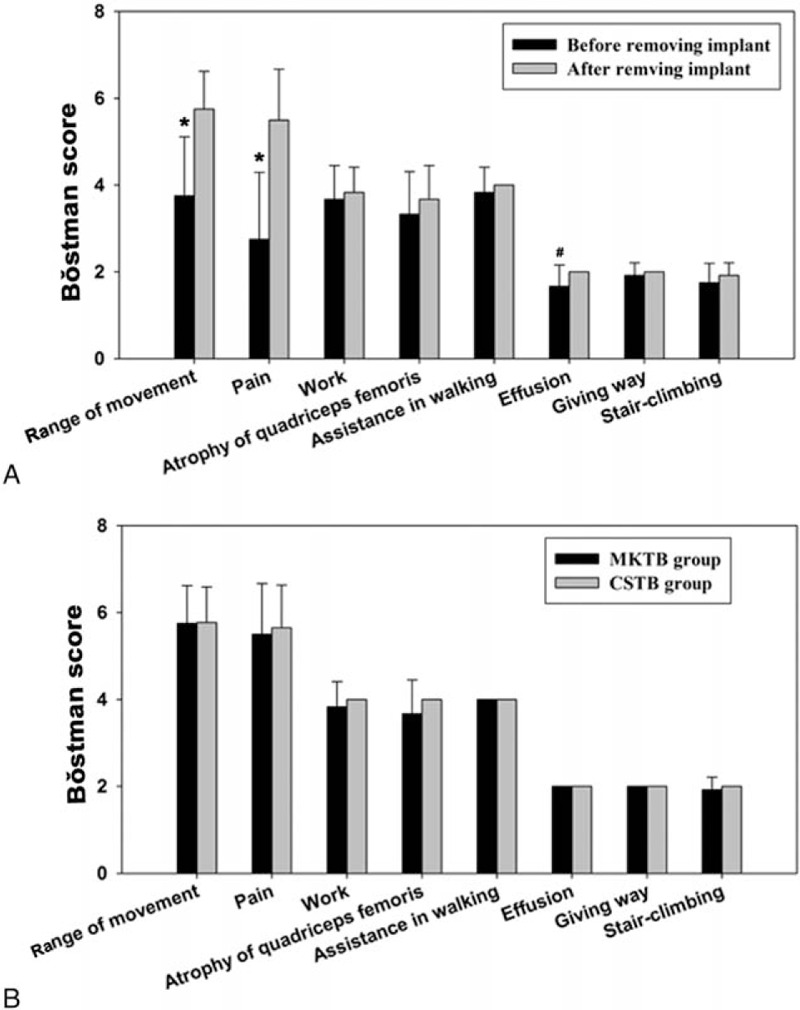
Bǒstman scores in the 2 groups. (A) Score in the MKTB group before and after removing implant. Compared to score after removing implant, ^∗^*P* < 0.01. There were no differences in the other evaluated parameters. (B) Scores of the MKTB group after removing implant compared with the CSTB group. There were no significant differences between the 2 groups (*P* *>* 0.05). CSTB = cannulated screw tension band, MKTB = modified Kirschner wire tension band.

At the time point before removing implant in the MKTB group (range 12–16 months, mean 13 ± 2.5 months), 20 patients (69%) in the MKTB group had a ROM of >120°, while the remaining 9 (31%) had a ROM of 90° to 120°. In the CSTB group, 24 patients (92%) had an ROM of >120°, whereas 2 patients (8%) had an ROM of 90° to 120°. Mean scores of ROM were 5.07 ± 1.41 in the MKTB group and 5.77 ± 0.82 in the CSTB group, showing a significant difference (*P* = 0.031) (Table 2). Twelve patients in the MKTB group underwent implant removal, the mean ROM score was 5.75 ± 0.87, showing a significant difference compared to the scores before removing implant (3.75 ± 1.36) (*P* = 0.001) (Fig. 1A), and reached levels similar to those in the CSTB group (5.77 ± 0.82) (*P* = 0.948) (Fig. 1B).

**Table 2:**
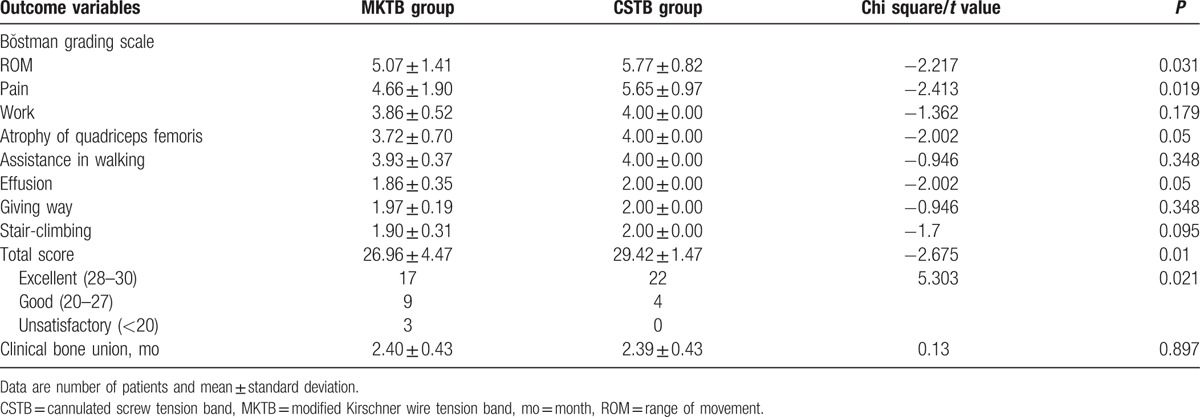
Clinical results of the 2 groups.

### 3.3 Joint pain

In the MKTB group, 18 patients (62%) had no or minimal pain on exertion, 9 (31%) had moderate pain, and 2 (7%) had pain in daily activity. Twenty-three patients (88%) in the CSTB group had no or minimal pain on exertion, and 3 (12%) patients had moderate pain. Mean scores for MKTB and CSTB groups were 4.66 ± 1.90 and 5.65 ± 0.97, respectively (Table 2), demonstrating a significant difference (*P* = 0.019). Mean pain scores in the MKTB group after removing implant (5.5 ± 1.17) increased compared with scores (2.75 ± 1.54) before removing implant (*P* = 0.000) (Fig. 1A), and became almost equal to the scores (5.65 ± 0.98) of the CSTB group (*P* = 0.674) (Fig. 1B).

### 3.4 Ability to work

In the MKTB group, 27 patients continued to pursue their original work but 2 patients changed from their original work to light work because of joint pain and limited movement; of these 2 patients, 1 returned to their former job after removing implant. In the CSTB group, all patients returned to their original work. The scores were 3.86 ± 0.52 for the MKTB group and 4.00 ± 0.00 for the CSTB group, showing no significant difference (*P* *=* 0.179) (Table 2). In the MKTB group, mean scores after removing implant (3.83 ± 0.58) were higher than those before removing implant (3.67 ± 0.78) with no significant difference (*P* *=* 0.339) (Fig. 1A). There were no differences between the MKTB group after removing implant and the CSTB group (*P* *=* 0.143) (Fig. 1B).

### 3.5 Atrophy of quadriceps femoris

Atrophy was assessed by measuring the difference in thigh circumference 10 cm proximal to the patella between the affected leg and the contralateral leg. Compared with the contralateral thigh, 4 patients in the MKTB group had a mean 14-mm difference, and in 2 this improved after removing implant. No atrophy of the quadriceps femoris was found in the CSTB group. The scores in the MKTB and CSTB groups were 3.72 ± 0.70 and 4.00 ± 0.00 respectively, showing a significant difference (*P* = 0.05) (Table 2). In the MKTB group, mean scores after removing implant (3.67 ± 0.78) were higher than those before removing implant (3.33 ± 0.98) showing no significant difference (*P* *=* 0.166) (Fig. 1A). There were no differences in the scores between the MKTB group after removing implant and the CSTB group (*P* *=* 0.166) (Fig. 1B).

### 3.6 Daily walking

In the MKTB group, 1 patient walked with the help of a cane part of the time, and 1 patient felt giving way sometimes, which disappeared after removing implant. Three patients felt disturbing when stair-climbing, but this symptom disappeared in 2 cases and improved in 1 case after removing implant. No functional disorders were seen in the CSTB group. In the MKTB and CSTB group, the scores for assistance in walking were 3.93 ± 0.37 and 4.00 ± 0.00, the scores for giving way were 1.97 ± 0.19 and 2.00 ± 0.00, and the scores for stair-climbing were 1.90 ± 0.31 and 2.00 ± 0.00, respectively, showing no significant differences (*P* > 0.05) (Table 2). For the MKTB group after removing implant, the scores for the aforementioned variables were 4.00 ± 0.00, 2.00 ± 0.00 and 1.92 ± 0.29, respectively, with no significant differences compared to the scores before removing implant or the scores of the CSTB group (*P* > 0.05) (Fig. 1).

### 3.7 Joint effusion

Four patients in the MKTB group (13.7%) had joint effusion during joint movement, which improved after removing implant. No joint effusion was reported in the CSTB group. The scores in the MKTB and CSTB groups were 1.86 ± 0.35 and 2.00 ± 0.00, respectively, showing a significant difference between them (*P* = 0.05) (Table 2). The scores for the MKTB group were 2.00 ± 0.00 after removing implant, showing a significant difference compared to the score before removing implant (1.67 ± 0.49) (*P* = 0.039) and equal to the score of the CSTB group (2.00 ± 0.00) (Fig. 1).

### 3.8 Total Bǒstman scores

**Figure 2:**
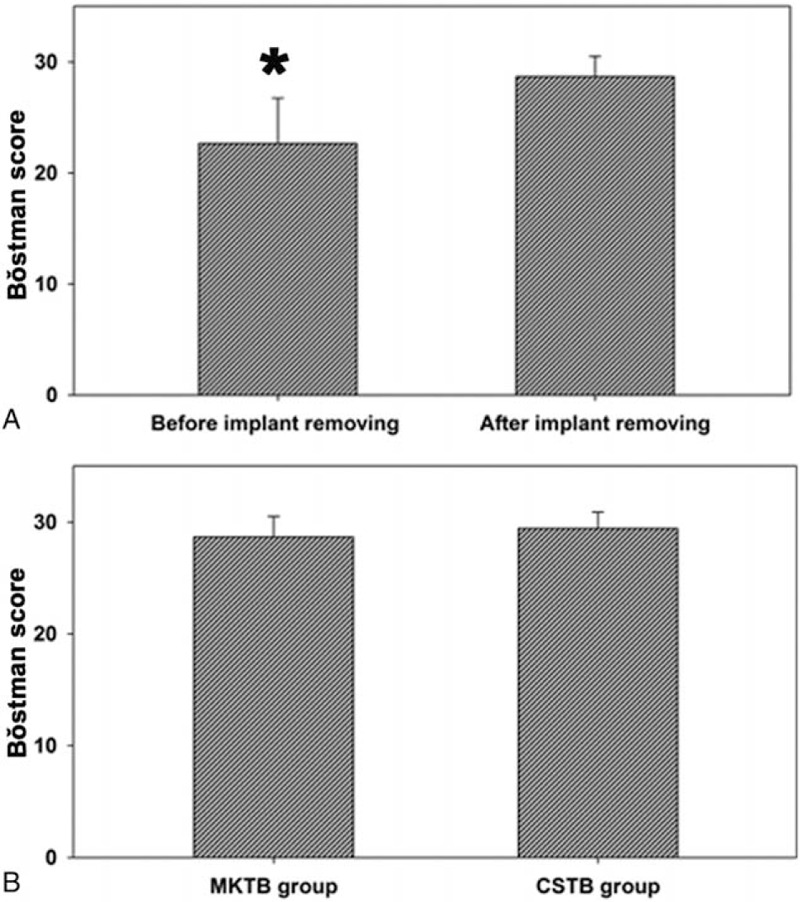
Total Bǒstman score. (A) Compared to score after removing implant in the MKTB group, ^∗^*P* < 0.01. (B) Scores of the MKTB group after removing implant compared with the CSTB group. No differences were detected between the 2 groups (*P* > 0.05). CSTB = cannulated screw tension band, MKTB = modified Kirschner wire tension band.

**Figure 3:**
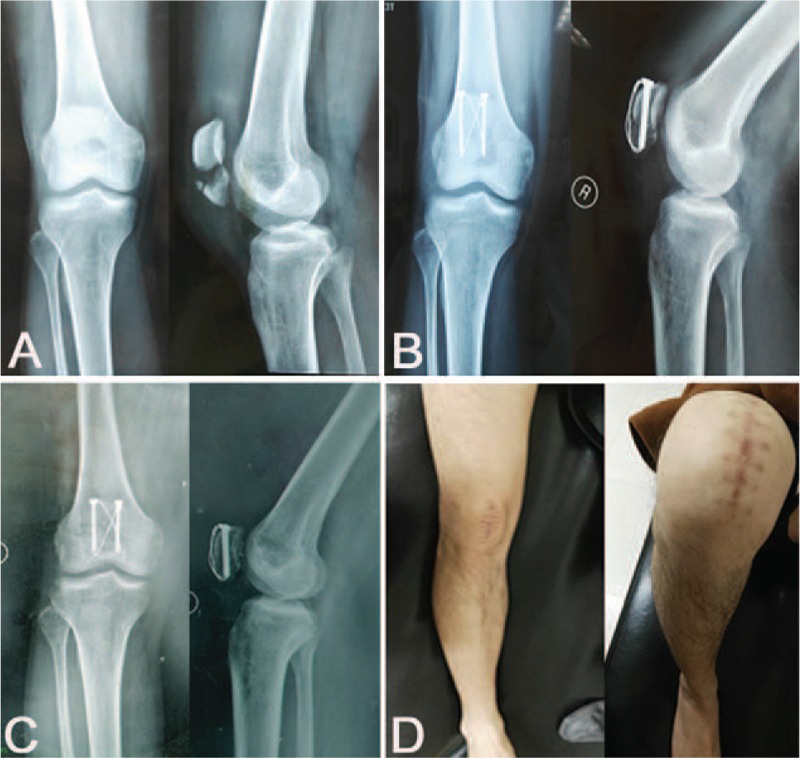
A 48-year-old male patient with right transverse patella fracture underwent CSTB fixation. X-ray film showing transverse patella fracture (A). Postoperative film showing anatomical reduction of the fragments and stable fixation (B). No loosening and displacement of the cannulated screw and steel wire 16 months after operation (C). Excellent knee functions (D). CSTB = cannulated screw tension band.

**Figure 4:**
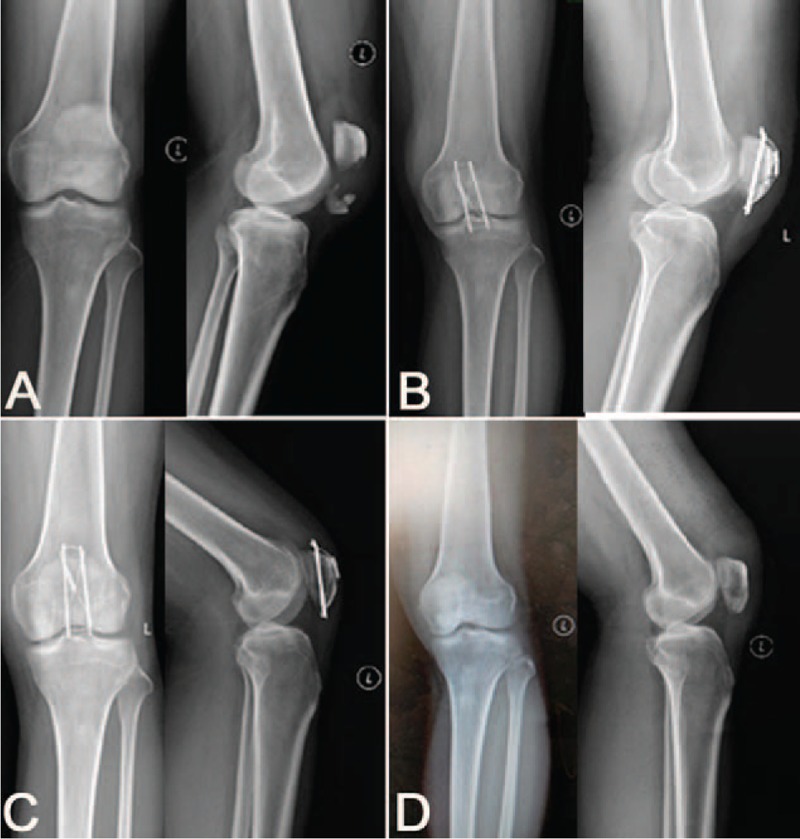
A 36-year-old female patient with left transverse patella fracture underwent MKTB fixation. X-ray film showing transverse patella fracture (A). Postoperative film showing stable fixation of the fragments (B). The fractures got bone union; however, the patient complained of irritating pain caused by relative long tail of the K-wires (C). The pain disappeared after removing implant, showing improvement of the knee function (D). K-wires = Kirschner wires, MKTB = modified Kirschner wire tension band.

At the follow-up evaluation before removing implant in the MKTB group, according to the Bǒstman grading system, 17 patients (59%) had an excellent result, 9 (31%) had a good result, and 3 (10%) had a fair result. In the CSTB group, 22 (85%) patients had an excellent result and 4 (15%) had a good result. A significant difference was found in the rate of excellent outcomes between the 2 groups (*P* = 0.021). After removing implant in the MKTB group (12 patients), 9 patients (75%) had an excellent result and 3 (25%) had a good result, showing a significant difference in the rate of excellent outcomes compared to that before removing implant (9 patients had good results, and 3 had fair results) (*P* = 0.001), but no difference compared to the CSTB group (22 patients had an excellent result and 4 had a good result) (*P* = 0.631) (Table 2). Mean total Bǒstman scores of the MKTB and CSTB groups (26.97 ± 4.47 and 29.42 ± 1.47, respectively) showed a significant difference at the follow-up evaluation before removing implant in the MKTB group (*P* *=* 0.01) (Table 2). Total Bǒstman scores in the MKTB group after removing implant (28.67 ± 1.83) were higher than that before removing implant (22.67 ± 4.05) (*P* = 0.001) (Fig. 2A). No differences were detected between the MKTB group and the CSTB group after removing implant (*P* *=* 0.224) (Fig. 2B). Figures 3 and 4 show typical cases.

### 3.9 Complications

**Figure 5:**
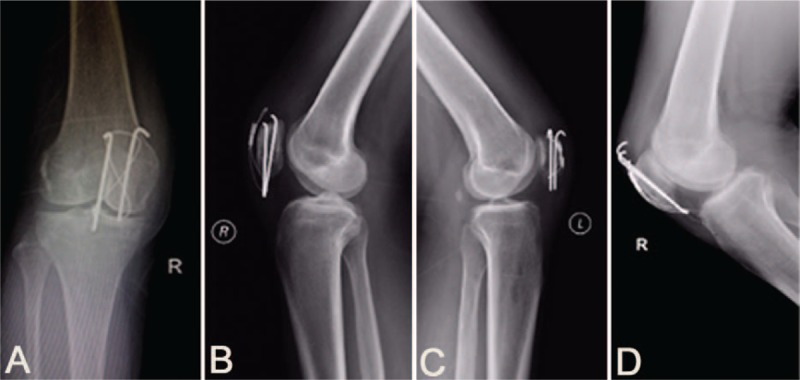
Displacement of hardware in the MKTB group. Displacement of 1 K-wire with no steel wire band loosening (A). Displacement of 2 K-wires with titanium cable loosening (B). Displacement of 1 K-wire with no titanium cable band loosening (C). Displacement of 2 K-wires with no titanium cable band loosening (D). K-wires = Kirschner wires, MKTB = modified Kirschner wire tension band.

According to clinical and radiographic results, all patients achieved bony union by 2.40 ± 0.43 months (range 2–3 months) in the MKTB group and 2.39 ± 0.43 months (range 2–3 months) in the CSTB group (*P* = 0.897) (Table 2). In the MKTB group, 11 patients felt moderate or severe pain during knee joint movement owing to soft tissue irritation caused by the K-wire ends, among which 4 patients had loosening and displacement of the hardware at a mean of 1 month after operation, and bone union was achieved using brace fixation (Fig. 5). The symptoms, such as irritating pain and restricted movement, improved or disappeared after removing implant. In the CSTB group, no severe pain occurred; other complications, such as implant loosening and displacement, also did not occur.

## 4 Discussion

It is well known that displaced transverse patella fractures require surgical intervention, and MKTB is the widely accepted technique for treating this lesion.^[8]^ In the MKTB technique, 2 parallel K-wires are longitudinally passed through the reduced fracture segments and a figure-of-eight tension band is applied to secure fracture fixation, the ends of the K-wires are then twisted and buried in the peripatellar soft tissue.^[8]^ Despite biomechanical and clinical studies demonstrating that the MKTB technique provides relatively stable fixation for transverse patellar fractures, deficiencies have been reported with this technique, mainly implant loosening, displacement, and irritating pain, which have an impact on limb rehabilitation, and potentially have a deleterious effect on functional outcomes.^[6,9]^ In order to avoid these problems associated with the MKTB technique, we used a CSTB to treat transverse patellar fractures and compared the clinical results of these 2 surgical techniques.

Based on the B**ǒ**stman scoring system, we compared postoperative knee joint function between the 2 groups. The results showed that the knee pain score, quadriceps atrophy score, and ROM score in the CSTB group were higher than the MKTB group at the follow-up evaluation before removing the implant, and a higher total Bostman score and excellent results characterized the CSTB group. According to a further analysis of each evaluation index of the Bǒstman scores, the knee functions differed between the 2 groups and were mainly associated with the irritating pain caused by hardware in the MKTB group. For the patients in the MKTB group with knee joint pain, functional limitation of the knee was improved after removing the implant owing to the pain relief, and the total B**ǒ**stman score and excellent results also increased and nearly reached the level of the CSTB group. Based on the aforementioned analysis, irritating pain caused by K-wires mainly account for the relatively poor knee functional recovery in the MKTB group.

In this study, 38% of patients in the MKTB group experienced mild to severe pain that affected knee joint function. This pain was caused by the relatively long length of the K-wires or their displacement which could irritate the extensor mechanism during knee flexion. Painful hardware was the most common complication in the MKTB group, which occurred in 30.1% of patients, and tension band loosening and migration was the second major complication, seen in 11.5% of patients.^[9]^ One previous study showed that K-wire prominence and migration are the primary causes of skin irritation associated with this technique.^[10]^ Other studies showed that the displaced K-wires caused by implant loosening were another main cause of irritating pain, which has been reported to occur in 0% to 20% of surgically treated patellar fractures.^[11–13]^ One reason for implant loosening is that the surface of the K-wire is smooth, so loosening is sometimes inevitable, whereas another cause is the technique used which involves twisting the steel wire at only 1 site, which might lead to implant failure.^[6]^ In this study, 4 patients in the MKTB group had loosening of the K-wires and steel wire tension band, which may be caused by the aforementioned factors. In our study, implant loosening was found at a mean of 4 weeks after operation, and no postoperative displacement of the fracture segments occurred. The fractures achieved bone union under knee-bracing fixation.

This study showed that the CSTB group had higher scores than the MKTB group for ROM, pain, atrophy of the quadriceps femoris, and joint effusion, which arose from the method of performing the CSTB technique. For this technique, the primary compression effect at the fracture interface is achieved by 2 parallel cannulated lag screws, and the second compression is achieved by tightening the figure-of-eight wire band over the anterior patella, which is different from the MKTB technique.^[14]^ The probability of wire-cannulated screw loosening is very low owing to the dense cancellous bone of the patella and the fact that the distal end of the cannulated screw is threaded.^[6]^ The tail of the cannulated screw tightly attached at the superior pole of the patella, the screw threads are not exposed at the edge of the patella, and the steel wire is close to the surface of the patella after being tightened, all of which reduce the risk of skin irritation and postoperative activity discomfort.^[6]^ The above CSTB fixation techniques facilitated early knee movement, preventing muscular atrophy, and intraarticular adhesions. In our study, no obvious skin irritation caused by hardware occurred, and the pain scores were higher than in the MKTB group.

The rates of removing implant have been reported to range from 37 to 55% for transverse patellar fractures, mainly because fixation with K-wires is associated with irritating pain of the soft tissue caused by the K-wires.^[15,16]^ Lazaro et al^[17]^ reported a rate of 37% hardware removal due to prominent and symptomatic implants as a result of breakage or continuous soft tissue irritation. In our study, the rate of removing implant was 41% (12/29) for the MKTB group, after which knee function improved due to the pain relief, and total scores and the rate of excellent results increased and almost reached the level of the CSTB group. No implants in the CSTB group were removed as there were no reports of pain caused by the hardware.

In conclusion, compared with the MKTB technique, CSTB fixation is an effective surgical procedure for treatment of displaced transverse patellar fractures. Use of the CSTB technique, which did not result in irritating pain, led to a higher rate of excellent clinical results. Although functional limitation arising from hardwire pain was commonly associated with MKTB fixation, removing implant after fracture healing was able to significantly improve knee function. Our study had 2 limitations. It was a retrospective study and not randomized; the sample size was not large. Therefore, the results may be biased.
